# Orientation Polarization Spectroscopy—Toward an Atomistic Understanding of Dielectric Relaxation Processes

**DOI:** 10.3390/ijms23158254

**Published:** 2022-07-26

**Authors:** Friedrich Kremer, Wycliffe Kiprop Kipnusu, Martin Fränzl

**Affiliations:** Peter Debye Institute for Soft Matter Physics, Leipzig University, Linnestr. 5, 04103 Leipzig, Germany; kipnusu@physik.uni-leipzig.de (W.K.K.); martin.fraenzl@physik.uni-leipzig.de (M.F.)

**Keywords:** orientation polarization, broadband dielectric spectroscopy, infrared (IR) spectroscopy

## Abstract

The theory of orientation polarization and dielectric relaxation was developed by P. Debye more than 100 years ago. It is based on approximating a molecule by a sphere having one or more dipole moments. By that the detailed intra- and intermolecular interactions are explicitly not taken into consideration. In this article, the principal limitations of the Debye approximation are discussed. Taking advantage of the molecular specificity of the infrared (IR) spectral range, measurements of the specific IR absorption of the stretching vibration υ(OH) (at 3370 cm^−1^) and the asymmetric υ_as_(CH_2_) (at 2862.9 cm^−1^) are performed in dependence on the frequency and the strength of external electric fields and at varying temperature. The observed effects are interpreted as caused by orientation polarization of the OH and the adjacent CH_2_ moieties.

## 1. Introduction

**Figure 1:**
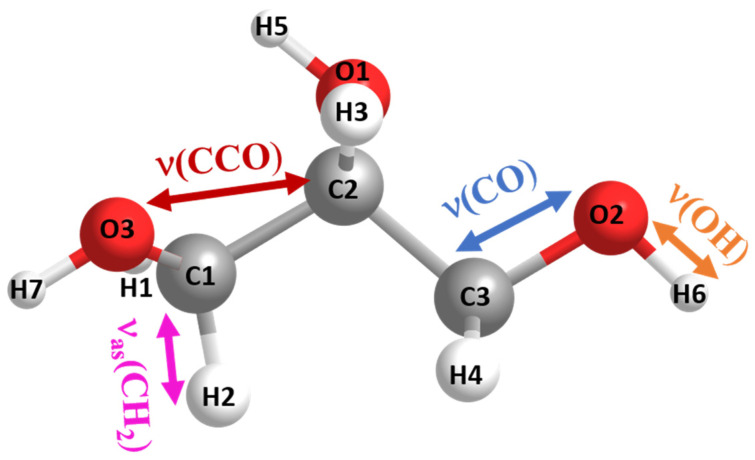
IR-active vibrations being involved in intra- and/or intermolecular interactions. The atomic charges (in electronic units) of the labeled atoms are according to [5]: O1: −0.581, O2: −0.585, O3: −0.585, C1: 0.182, C2: 0.055, C3: 0.182, H1: 0.026, H2: 0.026, H3: 0.04, H4: 0.026, H5: 0.396, H6: 0.396, and H7: 0.396. The calorimetric glass transition temperature Tg of glycerol is 185 K.

In referring to Einstein’s theory of Brownian motion [1], Debye developed the theory of orientation polarization [2,3,4] more than 100 years ago. It is based on approximating a molecule by a sphere having one or more dipole moments. In this approach, an atomistic understanding of the intra- and inter-molecular interactions is explicitly not considered. However, this can be only a rough approximation. If one considers, for instance, the widely studied glass-forming liquid glycerol as a model (Figure 1), one has to realize that it contains multiple intramolecular dipoles caused by partial charges of its atoms; furthermore, the intramolecular force-field parameters [5] vary widely from ~0.2 kcal mol^−1^ for torsional-type vibrations to ~300 kcal mol^−1^Å^−2^ for stretching potentials having additional temperature dependences, which are not known in detail.

A recent IR-based study [6] on a homologous series of polyalcohols, including glycerol, was conducted in a wide range of temperatures from far above to far below the calorimetric glass transition Tg. Thus, the potentials and hence the bond lengths of specific intramolecular and intermolecular interactions were determined. While the former has an expansion coefficient of (~0.1 pm/100 K) with only smooth changes, the latter shows a 30–40 times stronger response with pronounced kinks at Tg. It is to be expected that such heterogeneities on intramolecular and intermolecular scales are a general phenomenon in liquids and glassy systems.

Nowadays, modern experimental measurement techniques [7] are enabled to determine the fluctuations of polar molecules in a broad frequency range spanning typically more than 12 decades at widely varying temperatures. Thus, it became possible to study, for instance, the scaling of relaxation processes [8] in great detail. In comparing dielectric measurements performed along isobars and/or isotherms, the measured spectra is shown to collapse into each other if displayed as a function of a single variable, that is, a product of the inverse temperature and the density power with a material specific scaling exponent [9]. However, several examples [9,10,11,12] exist demonstrating that this scaling is only a rough approximation. In all these studies, only the mean relaxation rate is considered while the temperature-dependence of the relaxation time distribution function is neglected.

## 2. Theory of Operation for IR-Based Orientation Polarization Spectroscopy

**Figure 2:**
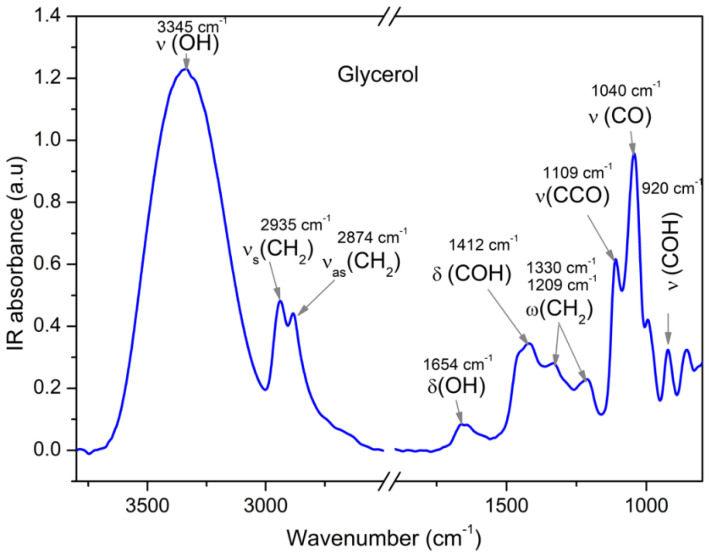
Characteristic IR-absorption bands [13] of glycerol, stretching vibration υ(OH), symmetric υ_s_(CH_2_) and asymmetric stretching vibration υ_as_(CH_2_), bending vibrations δ(OH) and δ(COH), and wagging vibration ω(CH_2_) and stretching vibrations υ(CCO), υ(CO), and υ(COH).

The infrared spectral range is known to have a “fingerprint” character for the intra- and intermolecular bonds of a molecule under study. Glycerol, for instance, has characteristic vibrations (Figure 2) representing the stretching of the (CO), the (OH), the (CH), and the (CCO) and the bending of the OH moieties. Applying external frequency dependent electric fields to a sample at widely varying temperatures and determining the field-induced IR-dichroism of specific absorption bands enables one to measure the orientation polarization of the respective molecular units separately.

An external electric field $\vec{E}$*_ext_* tries to orient a dipole $\vec{\mu }\;$against the thermal fluctuations of the molecular system as a whole [14]. The counterbalance between both results in an ensemble-averaged orientation polarization $\langle \vec{\mu }\rangle_{or}$ given by the Langevin equation

(1) $$\langle \vec{\mu }\rangle_{or}=\left|\vec{\mu }\right|\langle \cos\theta \rangle =\left|\vec{\mu }\right|L\;\left(x\right)=\frac{e^{x\;}+e^{-x}}{e^{x}-e^{-x}}=\coth x-\frac{1}{x}$$

where k_B_ is Boltzmann´s constant, *θ* is the angle between $\vec{\mu }\;\mathrm{and}\;\vec{E}$*_ext_*, *T* is the absolute temperature, and *x* = $\vec{\mu }$ $\vec{E}$*_ext_/*k_B_ *T*. In this approximation, the assumption is made that the different dipolar units fluctuate independently from each other. For the resulting orientation polarization, $\vec{P}$*_or_* holds

(2) $$\vec{P}{}_{\mathrm{or}}=n\;\langle \vec{\mu }\rangle_{or}(\varepsilon^{*}-1)\varepsilon_{o}\vec{E}{}_{\mathrm{ext}}$$

where *n* is the number density of dipoles $\vec{\mu },\;$ε^*^ is the complex dielectric function, and *ε_o_* is the permittivity of free space. If the dielectric function is determined by a *single* dielectric relaxation process having a relaxation time τ, then ε^*^ follows according to Debye theory of orientation polarization as

ε^*^ = *ε*′ − *iε*″ = *ε*_∞_ + (*ε_s_* − *ε*_∞_)/(1 + *iωτ*) (3)

where *ω* is the frequency of the external electric field $\vec{E}$*_ext_*(*ω*); *τ* is a characteristic relaxation time, and *ε_s_* and *ε*_∞_ are the values of the real part *ε*’ for *ωτ* << 1 and *ωτ* >> 1, respectively.

**Figure 3:**
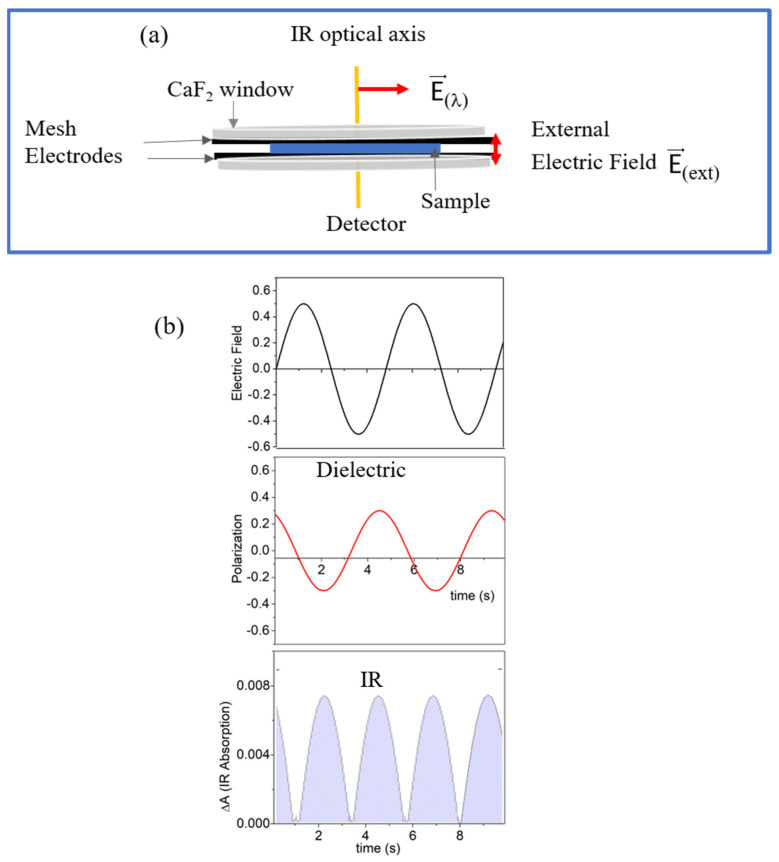
(**a**) Scheme of the sample cell: ${\vec{E}}_{\left(\lambda \right)}$ is the E-vector of the IR-light and $\vec{E}$ *_(ext)_* is the external electric field applied to the sample; (**b**) orientation polarization as measured by dielectric and IR spectroscopy in the second harmonic of the modulated absorption.

For a molecule, having a dipole-moment $\vec{\mu }$ and a transition moment $\langle \psi_{i}/\vec{\mu }{/\psi }_{e}\;\rangle$ in the quantum mechanical dipole approximation [15] the absorption *A* is proportional to

(4) $$A\sim \langle \psi_{i}/\vec{\mu }{/\psi }_{e}\rangle^{2}$$

where $\psi_{i}$ and $\psi_{e}$ are the wave functions of the initial and exited states. Assuming that these are not seriously changed by the application of an external frequency dependent electric field $\vec{E}$*_ext_* (*ω*), it is expected that the orientation polarization of polar moieties is also reflected in the square of the transition moment, which is proportional to the respective IR absorption (Figure 3). If the change in the absorption $\Delta A_{or}\left(\omega \right)$ is caused solely by orientation polarization,

(5) $$\Delta A_{or}\left(\omega \right)\sim \langle \psi_{i}/\langle {\vec{\mu \;(E_{ext.}\left(\omega ,\;T\right)}\rangle }_{or}/\psi_{e}\rangle^{2}$$

becomes frequency and temperature dependent. For isotropic systems, $\Delta A_{or}\left(\omega \right)$ is the same for positive and negative electric fields, and hence, it is evident in the second harmonic with respect to the frequency of the external electric field $\vec{E}$*_ext_*(ω). It is measured as modulation on a high absolute level of absorption in contrast to dielectric spectroscopy where one measures against zero. This means that phase sensitive detection is essentially required.

In order to estimate roughly the expected effects in absorption of a special IR-band, a molecule is assumed to have a dipole moment of 1 Debye; for a field strength of 10^5^ V/cm, one finds for the interaction energy between the external electric field, and the dipole a value of $\vec{µ}$ $\vec{E}$*_ext_~* 2 *×* 10^−22^ J. At a temperature of T = 300 K, the thermal energy k_B_*T* has a value of ~5 *×* 10^−21^ J, and hence x~$\vec{µ}$ $\vec{E}$*_ext_ /* k_B_ *T~*0.05. According to the Langevin Equation (1) for the induced averaged polar orientation, a value of 〈cosθ〉~0.017, and hence Δ*A*~0.0003 is obtained, which is small but well measurable with phase sensitive detection.

## 3. Experimental Section

**Figure 4:**
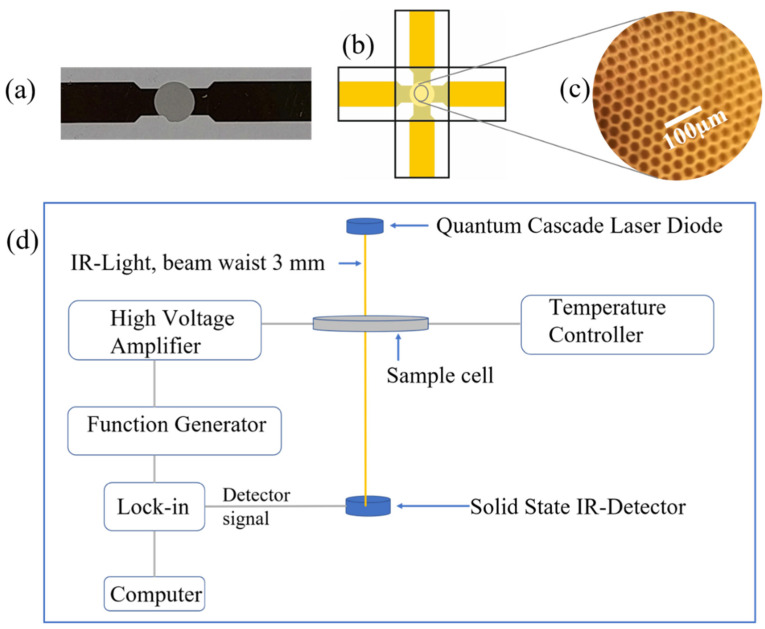
(**a**) Hexagonal evaporated mesh structure evaporated on an IR-transparent CaF_2_ substrate. (**b**) Scheme of the mesh electrodes in crossed arrangement; the evaporated electrodes are face to face with a separation of a few µm with the sample material in between. (**c**) Microscope image of the mesh structure with mesh size of 25 µm. (**d**) Scheme of the experimental setup; the quantum cascade laser diode emits a parallel beam (waist ~3 mm) of IR light transmitted through the sample cell and measured with a solid-state detector. Electric fields are applied to the sample by use of a function generator and a high-voltage amplifier. The second harmonic of the signal is analyzed by phase sensitive detection with a lock-in amplifier. The whole setup is fully computer-controlled.

The glycerol sample (purity 99%), having vapor pressure of <1 mm Hg (20 °C), refractive index of 1.47, and boiling point of 182 °C, was obtained from Sigma Aldrich and used as received. The sample was placed between CaF2 windows with evaporated mesh electrodes (Figure 4a–c). As spacer, a poly(ethylene terephthalate) (PET) foil of ~3 μm thickness was used. 

In order to realize the suggested experiments, the following experimental requirements have to be fulfilled: (i) the sample cell as a whole has to have an IR-transmission at least in the percent range; (ii) it must be possible to apply high-external electric fields (10^5^ V/cm–10^6^ V/cm) in a wide frequency (1–100 kHz); and (iii) it must be possible to apply temperature (100–400 K) range. To combine these features is not trivial. Evaporated nm thin metal electrodes as often used in Stark-effect studies [16,17,18,19,20] have a strong temperature dependence of the resistivity, thus excluding measurements at widely varying temperatures. This holds as well for semitransparent electrodes like ZnO etc.

A possibility to overcome the above outlined problems turned out to be hexagonal gold meshes (inner width of one mesh 26 µm, web width 8 µm, and thickness ~200 nm) electrodes (Figure 4a), which are evaporated on an IR-transparent CaF_2_ substrate (thickness 1 mm). For electrical insulation, the area with the mesh electrodes was covered by a ~150 nm protecting SiO_2_-layer deposited by chemical vapor deposition (CVD). The IR transmission of a single CaF_2_ window with evaporated mesh electrodes was ~40% at a wavelength of 2966.8 nm (3370.6 cm^−1^). In order to apply high external electric fields, the mesh electrodes were mounted in crossed orientation face to face (Figure 4b). A microscope image of two hexagonal electrodes is shown in (Figure 4c).

As radiation source quantum cascade laser diodes (S/N 2187/07-05, S/N 2186/07-27, Nanoplus, Germany) were employed emitting IR light either at a wavelength of 2966.8 nm (3370.6 cm^−1^) or 3493 nm (2862.9 cm^−1^); it was well directed with a beam waist of ~3 mm. The custom-made sample cell enabled temperature control in the range between 150–400K, with an accuracy of +/−0.2 K; external electric fields were provided by a function generator (Hewlett Packard 3312A) and a high-voltage amplifier (Pendulum A440D, maximum voltage +/−200 V (peak-peak) at frequencies between 1 Hz and 500 kHz) and applied to the sample by using the above-described hexagonal electrodes. As IR detector, a solid-state sensor (PDAV J5 Thorlabs) was used with buffer preamplifier. The signal was analyzed by a lock-in amplifier (SAR 550). The whole set up was fully computer controlled based on custom made LabVIEW software (Figure 4d).

## 4. Results and Discussion

**Figure 5:**
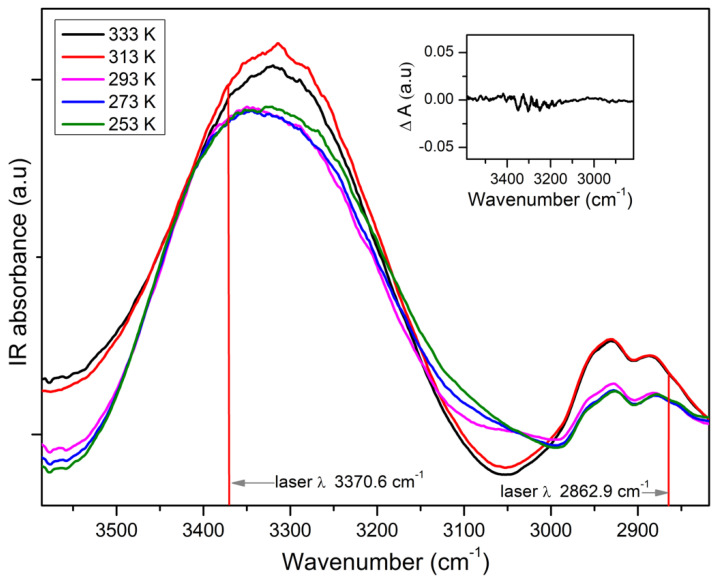
Characteristic IR-absorption bands of glycerol as measured with a Fourier-IR spectrometer at varying temperatures as indicated. The wavenumber of the quantum cascade laser diodes used in this study are marked. As spacer, a poly(ethylene terephthalate) (PET) foil of ~3 µm thickness was used; this results is for an applied voltage of +/−120 V in an electric field strength ~+/−0.4 MV/cm. The inset shows the difference IR spectrum between a sample measured with and without external electric field at 333 K.

Measuring with a Fourier transform spectrometer (Biorad FTS 6000), the IR absorption of glycerol at temperatures between 253 K and 353 K shows characteristic changes in the spectra, which are analyzed in detail in [6]. Comparing spectra measured with and without an external electric field of ~0.4 MV/cm and calculating the difference spectra delivers minute effects close to the noise level (inset in Figure 5).

**Figure 6:**
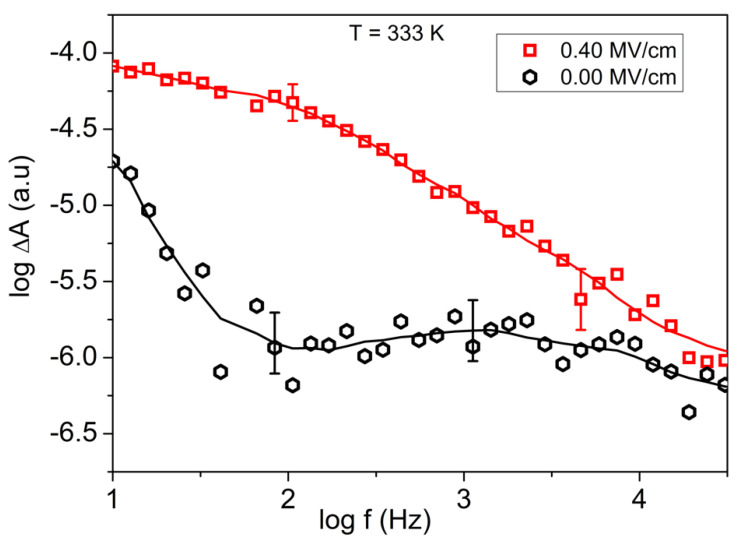
Example of the data analysis. The modulated signal (second harmonic) measured with the lock-in amplifier is displayed in dependence of the frequency of the external electric field. As spacer, a PET foil of ~3 µm thickness was used. This results for an applied voltage of +/−120 V in an electric field strength ~+/−0.4 MV/cm with a large uncertainty because the absolute sample thickness cannot be measured with sub-µm accuracy. The data are averaged, and for the resulting curve (solid lines), uncertainty bars are indicated. By division, the orientation polarization spectra are obtained as shown in Figure 7.

**Figure 7:**
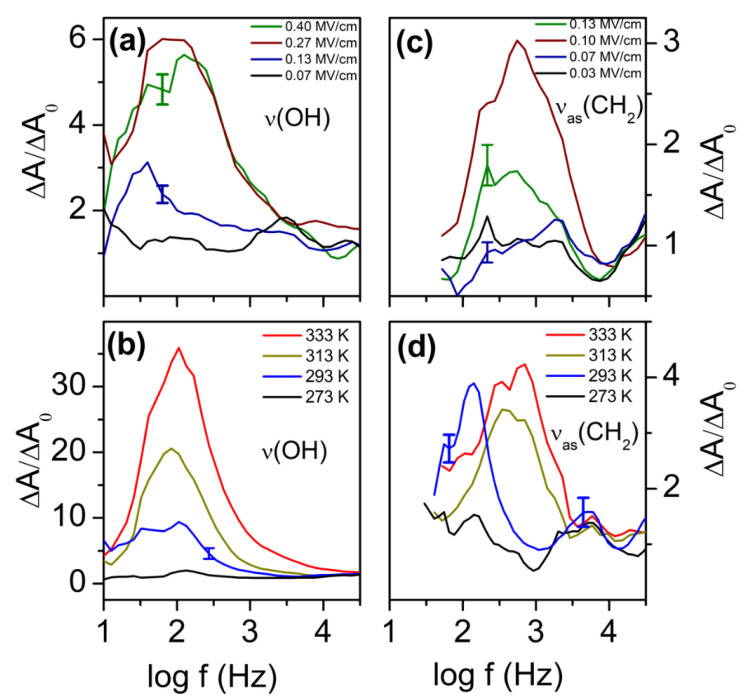
(**a**) Orientation polarization spectra $\Delta A_{or}\left(\omega \right)/\Delta A_{0}$ of the OH and CH_2_ moieties as measured by the lock-in amplifier (in the second harmonic) at the wavelengths of 2966.8 nm (3370.6 cm^−1^) and 3493 nm (2862.9 cm^−1^): (**a**,**c**) show the field strength; (**b**,**d**) show the observed temperature dependencies. As spacer, a PET foil of ~3 µm thickness was used. The result is an applied voltage of +/−120 V in an electric field strength ~+/−0.4 MV/cm with a large uncertainty because the absolute sample thickness cannot be measured with sub-µm precision. Within one figure, the sample thickness is assumed to remain nearly constant as confirmed by measurements of the IR-transmission.

**Figure 8:**
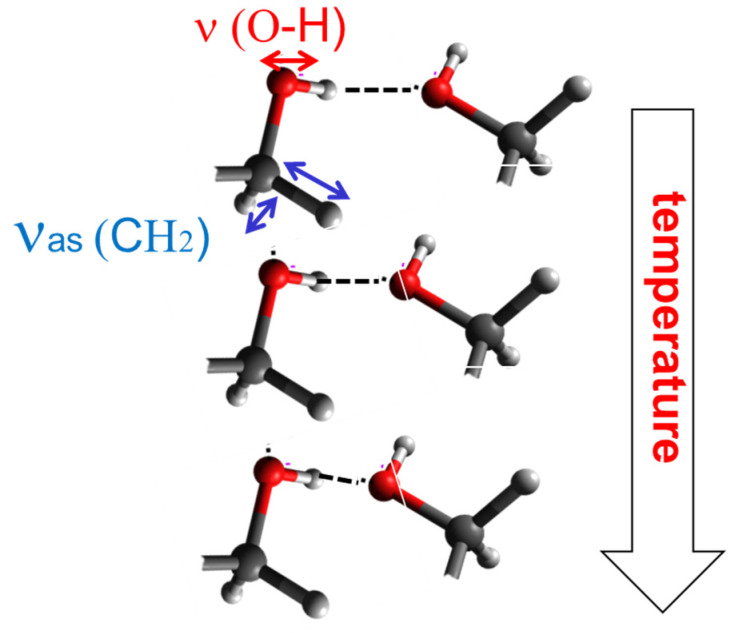
Scheme interpreting the observed specific orientation polarization for the O-H and CH_2_ moieties. The former has a dipole moment of 2.67 Debye; at temperatures > 283 K the O-H…O bonds are open, and the O-H dipole is subject to orientation polarization. With decreasing temperature, H-bonds are formed counteracting orientation fluctuations. The CH_2_ unit is nearly nonpolar, but due to the immediate neighborhood of the polar O-H moiety, it is coupled to its orientation fluctuations. With increasing H-bond, formation dies out as well.

The orientation polarization of the OH and the CH_2_ moiety is measured with the setup described in Figure 4. The modulation of the absorption $\Delta A_{or}\left(\omega \right)\;$is small but well measurable with phase sensitive detection (Figure 6). The noise level of the absorption $\Delta A_{0}\;$without external field is typically 10^−6^; it also has a frequency but no temperature dependence. By averaging over seven data points, curves typically are obtained having characteristic uncertainty bars. By calculating the ratio $\frac{\Delta A_{or}\left(\omega \right)}{\Delta A_{0}},\;$the orientation polarization spectra are obtained. Their frequency dependence for the OH and the CH_2_ units is shown in Figure 7a,c for varying strengths of the external electric field $\vec{E}$*_ext_*. The temperature dependence is shown in Figure 7b,d. The effect for the CH_2_ moiety is weaker than for the OH unit; this is caused by the much smaller dipole moment of the latter. The reorientation of this unit is presumably also influenced by a coupling to the adjacent OH group. With decreasing temperature H-bonds are formed, which hinder the fluctuation of both the OH and the adjacent CH_2_ moieties (Figure 8). The observed orientation polarization of the OH and CH_2_ moieties is much faster than the relaxation rate of the dielectrically measured orientation polarization. This reflects the fact that, in the latter, the relaxation of the molecule as a whole is measured while in the former, the orientation polarization of two molecular subunits OH and CH_2_ is monitored.

## 5. Perspectives

A severe limitation of the present study is the restriction by only two IR-wavelengths. However, IR-laser systems tunable in the entire “fingerprint region” are recently available, albeit still expensive. Using the methodology presented in this study, combined with those novel IR-sources, will enable one to unravel the orientation polarization of the different moieties of a molecular system and their mutual interactions, including nonpolar groups. Furthermore, by measuring in a wide temperature range the atomistic potentials determining the specific orientation, polarization can be determined. In summary, orientation polarization spectroscopy is the consequent development to unravel dielectric relaxation processes on atomistic scale.

## Data Availability

The data presented in this article are available from the corresponding author on request.

## References

[1] Einstein A. (1906). Zur Theorie Der Brownschen Bewegung. Ann. Der Phys..

[2] Debye P. (1912). Einige Resultate Einer Kinetischen Theorie Der Isolatoren. Phys. Z.

[3] Debye P. (1913). Zur Theorie Der Anomalen Dispersion im Gebiete der langwelligen elektrischen Strahlung. Verhandl. Deut. Phys. Ges..

[4] Debye P.J.W. (1929). Polare Molekeln (Polar Molecules).

[5] Chelli R., Procacci P., Cardini G., Valle R.G.D., Califano S. (1999). Glycerol Condensed Phases Part I. A Molecular Dynamics Study. Phys. Chem. Chem. Phys..

[6] Gabriel J.P., Tress M., Kossack W., Popp L., Kremer F. (2021). Molecular Heterogeneities in the Thermal Expansivity of Polyalcohols. J. Chem. Phys..

[7] Schönhals A., Kremer F., Kremer F., Schönhals A. (2003). Broadband Dielectric Measurement Techniques (10^−6^ Hz to 10^12^ Hz). Broadband Dielectric Spectroscopy.

[8] Kremer F., Loidl A., Kremer F., Loidl A. (2018). The Scaling of Relaxation Processes—Revisited. The Scaling of Relaxation Processes.

[9] Grzybowski A., Paluch M., Kremer F., Loidl A. (2018). Universality of Density Scaling. The Scaling of Relaxation Processes.

[10] Win K.Z., Menon N. (2006). Glass Transition of Glycerol in the Volume-Temperature Plane. Phys. Rev. E.

[11] Grzybowski A., Grzybowska K., Zioło J., Paluch M. (2006). Correlations between Isobaric and Isochoric Fragilities and Thermodynamical Scaling Exponent for Glass-Forming Liquids. Phys. Rev. E.

[12] Pawlus S., Grzybowski A., Kołodziej S., Wikarek M., Dzida M., Góralski P., Bair S., Paluch M. (2020). Density Scaling Based Detection of Thermodynamic Regions of Complex Intermolecular Interactions Characterizing Supramolecular Structures. Sci. Rep..

[13] Salehpour S., Dubé M.A. (2012). Reaction Monitoring of Glycerol Step-Growth Polymerization Using ATR-FTIR Spectroscopy. Macromol. React. Eng..

[14] Schönhals A., Kremer F., Kremer F., Schönhals A. (2003). Theory of Dielectric Relaxation. Broadband Dielectric Spectroscopy.

[15] Haken H., Christoph Wolf H., Haken H., Christoph Wolf H. (1995). The Quantum-Mechanical Treatment of Rotational and Vibrational Spectra. Molecular Physics and Elements of Quantum Chemistry: Introduction to Experiments and Theory.

[16] Shigeto S., Hiramatsu H., Hamaguchi H. (2006). Structure and Dipole Moments of the Two Distinct Solvated Forms of P-Nitroaniline in Acetonitrile/CCl4 As Studied by Infrared Electroabsorption Spectroscopy. J. Phys. Chem. A.

[17] Wang W.-C., Shigeto S. (2011). Infrared Electroabsorption Spectroscopy of N,N-Dimethyl-p-Nitroaniline in Acetonitrile/C2Cl4: Solvation of the Solute and Self-Association of Acetonitrile. J. Phys. Chem. A.

[18] Takashima K., Furukawa Y. (2015). Vibrational Stark Effect of 9-Cyanoanthracene Dispersed in a Poly(Methyl Methacrylate) Film. Chem. Phys. Lett..

[19] Schneider S.H., Boxer S.G. (2016). Vibrational Stark Effects of Carbonyl Probes Applied to Reinterpret IR and Raman Data for Enzyme Inhibitors in Terms of Electric Fields at the Active Site. J. Phys. Chem. B.

[20] Nguyen H.H., Loukianov A.D., Ogilvie J.P., Abramavicius D. (2020). Two-Dimensional Electronic Stark Spectroscopy of a Photosynthetic Dimer. J. Chem. Phys..

